# Attitudes Expressed in Online Comments about Environmental Factors in the Tourism Sector: An Exploratory Study

**DOI:** 10.3390/ijerph15030553

**Published:** 2018-03-19

**Authors:** Jose Ramon Saura, Pedro Palos-Sanchez, Miguel Angel Rios Martin

**Affiliations:** 1 Department of Business and Economics, Faculty of Social Sciences and Law, Rey Juan Carlos University, Paseo Artilleros s/n, Madrid 28032, Spain; 2 Department of Business Organization, Marketing and Market Research, International University of La Rioja, Av. de la Paz 137, 26006 Logroño, Spain; ppalos@unex.es; 3 Department of Financial Economy and Operations Management, Faculty of Economics and Business, University of de Sevilla, Av. de Ramon y Cajal, 1, Sevilla 41004, Spain; rios@us.es

**Keywords:** environment, sentiment analysis, Nvivo, twitter, textual analysis

## Abstract

The object of this exploratory study is to identify the positive, neutral and negative environment factors that affect users who visit Spanish hotels in order to help the hotel managers decide how to improve the quality of the services provided. To carry out the research a Sentiment Analysis was initially performed, grouping the sample of tweets (*n* = 14459) according to the feelings shown and then a textual analysis was used to identify the key environment factors in these feelings using the qualitative analysis software Nvivo (QSR International, Melbourne, Australia). The results of the exploratory study present the key environment factors that affect the users experience when visiting hotels in Spain, such as actions that support local traditions and products, the maintenance of rural areas respecting the local environment and nature, or respecting air quality in the areas where hotels have facilities and offer services. The conclusions of the research can help hotels improve their services and the impact on the environment, as well as improving the visitors experience based on the positive, neutral and negative environment factors which the visitors themselves identified.

## 1. Introduction

### 1.1. Global Overview of Tourism Trends

Over the past few years, the use of new technologies has led to changes in the environmental, cultural, economic and social sustainability sectors from how they were at the beginning of the 21st century [1,2]. Tourism and the activities linked to its growth have become a key part of the world economy and have also made local populations suffer the consequences of the impact of these activities on the environment [3].

Activities for tourism provide important amounts of income and jobs, promote knowledge of other cultures and conserve cultural and natural heritage, as well as investing in local infrastructure [4]. These activities provide both economic and social benefits. However, not everything is positive as some styles of tourism and certain recreational activities can lead to the destruction of habitats, the deterioration of the landscape and competition for scarce resources and services (fresh water, territory, energy, wastewater treatment, etc.) [5].

In addition, local populations may lose their traditional way of life as a consequence of these activities and could also become excessively dependent on the income generated by tourism [6]. The increase in prices associated with tourism can also negatively affect the local population, which runs the risk of losing land, houses, shops and services for the construction of hotels, hostels, rural houses or tourist services [7]. These problems are worsened by the concentration of tourist activity in relatively short holiday periods and in certain limited areas. These areas may also be subject to environmental pressures from other economic activities such as agriculture, fishing, and industrial development or from the increasing resident population. More than other sectors, tourism and recreational activities depend on the quality of the natural and cultural environment for their long-term success.

It can be seen therefore, that tourism can affect the natural environment to the point of endangering its own existence. To stop this, it is important to identify the environment-related factors which hotel users detect during their stays at hotels. These users are aware of the problems and the impact of tourism on the environment [8,9]. It is important to draw attention to the fact that the development of new technologies, and especially social networks has caused important changes to the tourism sector [6]. One great change is that people who stay at hotels can easily make comments on the Internet and provide information about their experience and the quality of their visit [9,10].

Like Hung, Kuo and Lin [6] the research of Bifet and Frank [7] analyzed the posts made on social network by companies in one sector. The results of the research show that the tone and narrative used in the post are directly associated to the interest shown in the post by both mobile device and desktop social networks users, which allows for the identification of the key factors which should be used to establish company strategies [11,12,13,14]. For some time, social networks and understanding their use has been of interest to researchers in different sectors, such as environmental, cultural, economic and social sustainability. In the work of Kwon [15,16,17], in which a qualitative approach is taken to determine the attitudes of older people, or the work of Honeycutt et al. [18] in which a textual analysis is performed to determine and understand the short- and long-term impacts in the field of environmental science and public health. 

### 1.2. Literature Review 

The interest that researchers have in identifying environmental factors which affect the users’ experiences when staying at hotels must also be considered. The interest of researchers in this area is shown in various research papers, such as those of Hussain and Singh [19], in which the research aims to discover the attitudes and behaviors of hotel users concerning sustainability. The work of Suanmali [20] is also concerned with detecting the factors that affect tourists’ satisfaction with the Northern Part of Thailand, using an empirical study relating to the environment.

In the work of Kaltenborn et al. [21], tourists’ attitudes are investigated concerning the environmental, social and managerial attributes of the Serengeti National Park, concluding with the identification of factors that affect hotel users concerned about the environmental impact of hotel activities. The work of Bruyere [22] aims to identify rural hotel users’ feelings about the benefits of exploiting rural areas for tourism and then suggest how the management of rural hotels can use these findings. Eliam and Trop [23,24] research users’ opinions on activities that affect the environment, identifying the main points which concern travelers.

In order to identify the factors that affect Spanish hotel users during their stay, we compiled a set of factors that affect the environment (see Table 1). It is important to identify the environment factors detected by the users of Spanish hotels as these users are already aware of the problems and the impact of tourism on the environment. Therefore, the experiences and feelings of these users when identifying environment factors of the tourism industry are very valuable. 

It is also useful to remember that the world tourism sector keeps growing [25]. In 2016, 1235 million tourists traveled to foreign countries, which means some 46 million more than the previous year, according to the World Tourism Organization (WTO) [26,27]. This is the seventh consecutive year of increases. However, the growth rate has slowed down as in 2016 the increase was 3.9%, whereas in previous years the rise has been around 4.6%. This means that last year there were 300 million foreign tourists more than in 2008 [28,29,30].

If we look at 2017 WTO world tourism figures, the number of visitors at destinations all around the world shows that these was a large demand for international tourism in the second half of 2017. Worldwide, international tourist arrivals increased by 6% compared to the same semester of the previous year, far surpassing the sustained and constant growth trend of at least 4% observed since 2010 [31]. These figures give the first six months of 2017 the best semi-annual results obtained in the last seven years. The results are related to the strong growth registered in many destinations and the continuing recovery in those that had registered falls in previous years. Of all WTO regions, growth was greatest in the Middle East (+9%), Europe (+8%) and Africa (+8%), followed by Asia and the Pacific (+6%) and then the Americas (+13%) [27].

In addition, the WTO research concludes that the growth of arrivals was driven by the demand for outbound tourism from the main sourcing markets. In particular, Canada, China, the Republic of Korea, Spain (object of our investigation), the United States, France and the United Kingdom, which have continued to report strong expenditure growth of outbound tourism.

### 1.3. The Context of Hotel Tourism in Spain

When studying the state of tourism in Spain, which is the geographical area on which this study is centered, we found that Spain received 75.6 million tourists in 2016. These figures are 10.3% higher than for 2015. In the last month of 2016, Spain was visited by 4 million foreign tourists, a figure that means an advance of 13.3% on the previous year. In Spain, this sector is a very important part of the national economy, contributing to nearly 11% of GDP (Gross Domestic Product) [27]. 

Tourists have also increased their spending to 77,000 million Euros. Tourists in Spain usually stay for between 4 and 7 nights. The main tourist destination in Spain was Catalonia. With 18 million tourists, it received 4% more visitors than in 2015. Next were the Canary Islands (with 13.3 million and an increase of 13.2%) and Illes Balears (with 13 million which means an increase of 11.9%) [27,28].

To understand the relevance of the analysis of the best hotels in Spain, we can see the results of FRONTUR’s research in Table 2. This shows that the number of tourists using paid accommodation as their main type of accommodation increased by 15.1% annually in December. In this market, hotel accommodation increased by 11.2% and rented housing increased by 49.2%. Unpaid accommodation showed an increase of 9.1%. Tourists housed in family or friends homes increased by 0.3% and those staying in self-owned housing by 42.5% [28].

In order to calculate the consequences tourism has on the environment it is also useful to see how these travelers arrived. The largest number of tourists to Spain arrived by air transport (December 2016) with almost 3.2 million arrivals, which represents an annual growth of 16.6%. By road, 0.1% more tourists arrived than in December 2015, while 41.3% more entered by rail and 8.8% more through sea ports, as can be seen in Table 3 [22].

This data emphasizes the importance that tourism has in the Spanish economy. In order to guarantee its future, companies and especially hotels, must identify the parts of their businesses that affect the environment [21,22].

One of the most important factors to be considered is the protection of the environment. This concept includes many points, not only landscapes and natural resources, but is also closely linked to the quality of the services which are offered and is a variable that constantly appears in the tourism sector [22].

This research can therefore be seen to be of interest to the management of hotels, who have to understand that the activities of their hotels and related services affect the environment and world sustainability. Consequently, it is important for them to improve their services by detecting if the hotel users considered that the environment is well treated or not during their stay at the hotel. 

Hotels should recognize the importance of this sector as a resource that represents the environment, so that it is not harmed in any way and therefore does not affect the current and future economic and social stability, which is a very important sector in the global economy. The future proposal for the tourism sector is based on quality and sustainable tourism which respects the environment [21]. Tourism and recreational activities depend far more than other sectors, on the quality of the natural and cultural environment for their long-term success. In this way, when a country which has attractive areas for tourism becomes an interesting destination for tourism and recreational activities, uncontrolled environmental impacts can jeopardize future benefits [22]. Therefore, tourism can affect the natural environment to the point of endangering its existence, hence the importance of identifying factors that affect the environment as a result of tourism activities in hotels.

In this sense, with the developments produced in sectors such as environmental, cultural, economic and social sustainability, this research is concerned with the identification of environment factors from the analysis of the Twitter users’ opinions for the 25 Hotels that won the Traveler’s Choice award from TripAdvisor in 2017. In this exploratory study, Twitter as a means of commenting on a topic, the opinions of hotel users and the identification of key environment factors that affect hotel visitors will be studied.

To complete this exploratory study a Sentiment analysis was carried out using an algorithm developed in Python by MonkeyLearn API (MonkeyLearn, San Francisco, CA, USA) [32] with which 14,459 tweets were grouped according to feeling (positive, negative or neutral) and then a textual analysis was performed on these tweets to identify the main environmental factors from the feelings shown using the qualitative analysis software Nvivo (QSR International, Melbourne, Australia). Afterwards, a linear correlation analysis and difference between means test were done using IBM SPSS version 24 (IBM, Chicago, IL, USA) and the analysis and results were presented. Finally, a discussions and conclusions section was written for the results obtained. 

## 2. Related Work

### 2.1. Machine Learning and Sentiment Analysis Approaches for Social Network Analysis

There are several researchers that have developed models based on machine learning for the analysis of social networks, the opinions that users have or to identify key factors related to a specific theme [33,34]. Supervised methods based on classification and categorization of key factors such as MaximumEntropy (MaxEnt) and Support Vector Machines (SVMs) are used for a combination of features to perform social network analysis with machine learning and research methods based on technology to identify the important factors of a research category [7,35,36,37]. These investigations can be based on keywords, ratings of feelings regarding a topic, semantic meaning, concepts and semantic theories, sentiment-topic features such as hashtags, retweets or points on social networks, and valuation identifiers for products and services on the Internet [38,39].

The research of Pak and Paroubek [40] presents an in-depth development of methodologies based on a study of Twitter. In general, semantic approaches based on sentiment analysis determine the occurrence of the key words, to which a statistical factor has been added. This factor determines the feeling [41] and is often used to perform analysis of positive and negative feelings and shows that Twitter can be an appropriate platform for researching factors of interest [42,43].

In the research by Zhang, Yun, Liang y Zhang [44] a semi-supervised dual recurrent neural network is proposed to prepare a Sentiment Analysis. This is similar to traditional neural networks and can be used to evaluate a set of data over a long period of time. This technique allows a more effective and efficient sentiment analysis to be carried out.

In Turney y Pantel [45], a recursive neural network is used to understand the meaning of particular comments. To achieve this, sentiment analysis identifies words and offers the semantic meaning for the particular topic of interest [46,47,48]. Neural networks are used to establish labels for each word and classify them according to predetermined criteria.

The research of Robinson [49] proposed a probabilistic model that is called Textual-based Information Diffusion and Evolution (TIDE), with which the evolution of different topics in social communities and their diffusion over time was measured. This method was based on Sentiment Analysis. The model extracts characteristics from the text of the comments made and implicitly captures them using standard ranges of the Gaussian field, as other authors have done [50,51,52,53].

Table 4 shows a summary of the main research using Sentiment Analysis and textual analysis combined with other methodologies based on machine learning and semantic analysis.

### 2.2. Textual Analysis

Textual analysis is a qualitative and exploratory procedure that determines the key factors of an event or object of study by grouping them into topic nodes. The Nvivo qualitative analysis software is one of the most relevant in this research category and has been used on numerous occasions in the last decade as a research method [55,56,57,58].

In Vázquez and Escamilla [52] a qualitative approach using the Nvivo software is taken to determine the attitudes of older people in order to perform a textual analysis on the contents of the results of the research. Ramirez-Andreotta et al. [53] also carry out a textual analysis to determine and understand the short- and long-term impacts of bio-monitoring and exposure of the participants in the study, in order to identify future factors for environmental justice using the Nvivo software.

In the work of Saito, Nakano and Kimura [59] a probabilistic matrix for the prediction of re-tweets based on textual analysis was developed. The social context of the relationships between the messages and their time latency were studied. Likewise, Jiang et al. [60] analyzed the fundamental factors that affect a concept called “re-tweetability” of each tweet when using a predictive filter based on collaboration between users. Textual analysis is used to determine and identify the most repeated factors of the study, and, based on these, determine the corresponding actions for the investigation.

In Table 5 the research using textual analysis can be seen, along with the main characteristics of qualitative analysis software such as Nvivo, which includes classification into nodes, categorization by topic, number of times a keyword is repeated and the type of keywords that are repeated. 

## 3. Conceptual Framework and Hypothesis Development

As we have already outlined, the interest of researchers over the last decades has been focused on the identification and analysis of key determinant factors in social networks with regard to a specific topic. Suanmali [24] detects the environment factors that affect the satisfaction of tourists by using an empirical study in the Northern Part of Thailand and links these factors to the actions carried out by hotels in the geographical area in which they are located. Kaltenborn [25] also investigates the attitudes of tourists to the environmental, social and managerial attributes of Serengeti National Park, concluding with the identification of factors that affect the interest of users and then defines and suggests actions that can be taken by the hotel sector to respect and conserve the environment. 

The environmental actions carried out by hotels are those actions related to the improvement of services based on social policies, the promotion of traditional products or local consumer goods, services related to the quality of the air or the mountain areas where the facilities are located, etc. Pak and Paroubek [40] carried out research in which a recursive neural network was used to understand what a particular content says and categorizes it with respect to the identification of specific terms. Pak and Paroubek [40] undertake a sentiment analysis to identify key words and offer the semantic meaning for each of the positive, negative or neutral links [42]. Based on the statements made above, it is proposed that:

**Hypothesis 1** **(H1).** The type of consumer experience in Spanish hotels (positive, negative or neutral) influences the environmental actions carried out by hotels.

As indicated previously by Bruyere [26], who identified the feelings of users who visited hotels in rural areas and how these influenced the users’ experiences in hotels in those areas. Eliam [27] also investigates user attitudes to the environment and identifies the key factors that were expressed.

Likewise, Moreno et al. [12] develops an enriched consumer recruitment system to increase the retention of users in campaigns for community schemes on social networks, classifying the key factors that was shared in the content and relating them to the research objective [61].

The factors that users consider to be relevant to the environment in hotels can determine their satisfaction with the services contracted in the hotel and can modify their enjoyment in a positive or negative way. The work of Roshan et al. [11] focuses on companies in different periods in order to categorize what the most important factors are when assessing the users’ experience with the companies. Roshan et al. [11] evaluate users experiences and opinions, and determine key factors from these [62]. Therefore, the following hypothesis is presented:

**Hypothesis 2** **(H2).** The environmental factors that users of Spanish hotels observe during their stays influence their experience at the hotel.

## 4. Methodology

The methodology used was firstly to perform a Sentiment Analysis on Twitter posts [7,33]. Using the results of this Sentiment analysis, a textual analysis was done to identify the key factors related to the environment [44,46]. Finally, bivariate linear correlation was used to demonstrate the statistical significance of the results [63] and then a difference of means test was done on the type of hotels, which were grouped according to their star ratings. This test was based on the Levene Test using the statistical analysis program IBM SPSS version 24.

### 4.1. Sample

As we have already indicated, the objective of this exploratory study is to identify the positive, neutral and negative factors related to the environment that affect the experiences of hotel users. These factors can help hotel management to improve their services and propose new social responsibility strategies for sustainability and respect for the environment such as, tourist activities which respect the environment or the promotion of activities that do not pollute the environment.

The research sample is composed of Spanish hotels ranked using TripAdvisor Traveler’s Choice Awards, which draws from more than 500 million opinions from travelers in Spain. Also, it is important to notice that Spain has become the second most visited country in world (82 million tourists visited Spain in 2018 according to data from the government of Spain). To identify the factors related to the environment, we analyzed the reviews that hotel users made on Twitter between 1 October 2016 and 1 October 2017 for the 25 hotels that won the Traveller’s Choice Awards. A total of *n* = 14,459 tweets for the 25 hotels that make up the sample were analyzed [64]. As we have already indicated, the research sample is made up of the Twitter profiles of the 25 hotels that won the TripAdvisor Traveller’s Choice Awards in 2017 and that have been classified according to:

- Active and official profile on Twitter
- Public profile
- Number of opinions in tweets
- Number of interactions with users

Appendix A shows the identification of the hotels under study in TripAdvisor and Twitter. 

### 4.2. Data Collection and Extraction

Data was collected using the Twitter API between 1 October 2016 and 1 October 2017. To carry out the Sentiment Analysis, we used the access algorithm in the MonkeyLearn API [32] that is written in Python and uses machine learning techniques to improve the levels of prediction and significance.

Firstly, the data was collected and classified using the Twitter API. Then, after having thoroughly trained the algorithm that does the Sentiment Analysis with part of the sample data, the entire database was analysed. The tweets for each hotel were divided into negative, positive and neutral groupings. Thirdly, a textual analysis was performed using the Nvivo 11 software (QSR International, Melbourne, Australia). The tweets were separated into nodes according to the feelings expressed (N_1_, N_2_ and N_3_) and then thematic nodes were identified to test N_4_, which is made up of the key environment factors.

### 4.3. Textual Analysis

The next step was to perform a textual analysis to identify the environment factors in the analyzed tweets. We used the Nvivo software to do this as it allows us to completely configure the Analysis for our test purpose [22].

The Nvivo software allows for classification by Nodes. The Nodes are configured as containers for the information which includes the evidence and has already been grouped beforehand [65,66,67]. It must be emphasized that the creation, design and exploration of nodes is a way to research pure data, in order to achieve higher quality descriptive and explanatory levels than could be reached without it [68]. Free nodes are data containers that can group ideas separately and that are not conceptually related to other nodes in an analysis. Branched nodes, are used to represent concepts grouped by categories which are logically linked and can be grouped hierarchically. The analysis results are then presented and are characterized using different indicators [69].

To do this we have established different nodes to identify the main environmental factors from the feelings examined with the nodes [70]. Firstly, a textual analysis was carried out on environmental factors in the tweets, classified as negative feelings (N_1_), secondly, with the tweets classified as neutral feelings (N_2_) and thirdly with the tweets classified as positive feelings (N_3_).

An exploratory analysis of each of the nodes allowed us to identify environment factors which were grouped into the node (N_4_) and then divided into three categories. Table 6 shows the classification of the nodes according to negative, neutral and positive environment factors [71].

## 5. Findings

### 5.1. Sentiment Analysis

In order to identify the environment factors observed by hotel users, the results of the textual analysis were analyzed for each of the nodes [72].

Firstly, the classifications which were used for the Sentiment Analysis carried out with machine learning are presented. These classify all of the users’ opinion tweets into positive, negative and neutral [73].

Next, in Table 7, the probability coefficients obtained from the classification of the Sentiment Analysis are presented with a summary of the content averages for each feeling. Then, the results of the textual analysis are presented for the nodes that subdivide each of the topics and factors that have been identified in the research.

The total number of analyzed tweets, *n* was 14,459. The average of published tweets was 657.22 and from the machine learning Sentiment Analysis the greatest probability percentage was 0.679 and the least was 0.555 [74].

**Figure 1:**
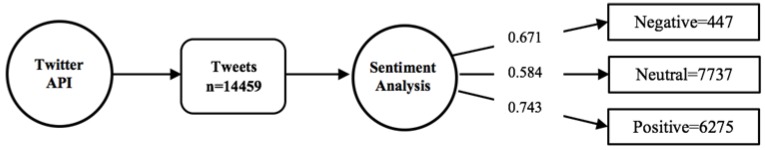
Classification of tweets according to feelings about the environment.

The probability percentages resulting from the Sentiment Analysis for each tweet classification can be seen in Figure 1. The probability percentage is a measure of accuracy, precision and recall of the samples in each category.

From all the analyzed tweets, 477 were negative, 7737 were neutral and 6275 were positive, which show that Twitter can be used as a platform to establish a relationship with the user [75]. 

### 5.2. Textual Analysis 

To identify the key environment factors in these user interactions, we carried out a textual analysis with Nvivo, for which each of the nodes of the textual analysis was divided into positive, negative and neutral nodes for the environment factors [76].

**Figure 2:**
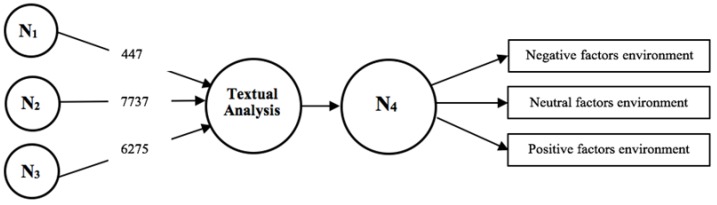
Relationship of Nodes and number of tweets for environment factors identification.

In Figure 2 below, we can the results of the global textual analysis after the Sentiment Analysis, and the environment factors which were tested [77].

The textual analysis of the environmental factors in N_1_ found the following results when analyzing the global opinions of users about their experience in the hotel [78]. Table 8 shows the Semantic analysis for negative environmental factors and Table 9 shows three negative tweets examples.

The negative factors in N_1_ show the users’ concerns about garbage collection, air pollution and the pollution of the hotels’ environment [76]

These results demonstrate the users concerns about negative environment factors. The weighted percentages do not exceed 0.06 for the negative content, so we can affirm that the hotel users are not highly concerned about these environmental factors [76]. 

The environment factors for N_2_, neutral factors, are shown in Table 10 below and Table 11 shows three neutral tweets examples.

The textual analysis of quality of service factors (N_2_) gave results showing how the users consider the products and services that the hotel makes available for planned trips and specialized offers.

Neutral environment factors were found, such as the importance of local products in local markets and shops, crafts from local potteries and artisans, traditional experiences such as visits to shelters, seminars and local monasteries that have not suffered changes over the years and finally a respect for nature, rivers and geographical features when constructing villages, along with the preservation of mountains and local roads. By using weighted percentages for N_2_, it can be seen that users give greater importance to N_1_. The highest weighted percentage for environment factors in the textual analysis was 0.40. Finally, the results of the textual analysis for environment factors of the 6275 tweets that make up the N_3_ node can be seen in Table 12. Also, Table 13 shows three neutral tweets examples.

A great variety of similar environment factors were found for N_3_. In particular, different contents about local tradition, customs, air quality, traditional ecology and the importance of nature were analyzed and evaluated.

The users valued positively the environment factors regarding hostels, routes, local products and shows. In addition, use showed positive relationships with the environments of dances and traditional customs, such as pastry making and viticulture. Another very important factor is the quality of the air, which can be linked to breathing problems, asthma and other diseases [73].

Traditional ecology is represented by factors such as salt pans, orchards and organic products. In general, nature is linked to concepts such as oasis, islands, mountains, rivers and paths. The weighted percentages of the textual analysis of N_3_ are between 0.39 and 0.09 in terms of positive factors related to the environment.

Table 14 shows all the environmental factors identified as a result of the Sentiment Analysis and subsequent textual analysis of the opinions and tweets of the hotel users that make up the sample. 

Table 11 summarizes the main environmental factors in the users’ opinions for the hotels of the 2017 Traveler’s Award and can be used to highlight the most important factors for the consumer. That is to say, the analysis of the results shows how the Spanish hotels users express their attitudes towards the environment factors which influence their experiences.

The results of this exploratory study can be used by hotel management to pay attention to the factors which are shown to be important to the guests. The positive factors that affect users visiting Spanish hotels are directly related to the activities carried out or managed by the hotels and which are associated with local traditions, air quality, customs, traditional ecology or nature. Hotel management can therefore organize activities that promote these factors to improve the users’ experience.

The results also show that the activities provided by the hotels with local and artisan products, traditional experiences and care for nature are factors that are neutral for visitors. However, the results of the exploratory study show that hotel management can use these results to improve their activities, initiatives and services, taking into account that the users regard garbage collection, atmospheric pollution and pollution as negative environment factors.

### 5.3. Linear Correlation Analysis and Difference between Means Tests

In order to show the statistical significances as an additional analysis of the investigation results, a bivariate linear correlation was made to check the possible relationships between the variables. The dependent variables were the number of followers on Twitter, and the negative, neutral or positive feeling of the tweets that were in the results [63]. The independent variables are the number of stars that the hotels under study have on TripAdvisor, the number of comments in the official Twitter profiles and the number of followers [64,65].

This test is used to check the possible relationship between two metric variables. The measurement is carried out using the linear correlation coefficient, which ranges between 1 and −1. If the value is 1 and positive, it indicates variation in the same direction for both variables. A negative value of 1 indicates variation in opposite directions, which means that when one of the variables increases, the other decreases. A value close to zero means that the variables behave independently. Therefore, correlation shows the extent to which two variables share a variation. To calculate the percentage of joint variation, the coefficient of determination is used, which is the square of the linear correlation coefficient. Correlations measure how variables or rank orders are related. The results of the correlations are shown in Table 15. 

High correlation between five variables can be seen: Number of Environment Tweets, Number of Followers, Negative Environment Tweets, Neutral Environment Tweets and Positive Environment Tweets, and also high significance (**) of two-by-two correlation coefficients. This can be between −1 (a perfect negative relationship) and +1 (a perfect positive relationship). A value of 0 indicates that there is no linear relationship.

The test for significance was unilateral probability, since the direction of association was Number of Environment Tweets towards the type of Tweets and Number of Followers towards Number of Tweets.

Correlations with significant correlation coefficients (*p* ≤ 0.05) are indicated with a single asterisk and those with (*p* ≤ 0.01) are identified by two asterisks.

The results show that there are significant correlations (*p* ≤ 0.01) between Number of Tweets and Negative Environment Tweets, Neutral Environment Tweet and Positive Environment Tweets. In all three cases correlation is positive and close to +1.

Also, there is significant correlation (*p* ≤ 0.01) between Number of Followers and Number of Environment Tweets. In addition, we can conclude that the higher the number of tweets in a hotel profile, the higher the number of negative tweets (Pearson correlation = 0.648, *p* ≤ 0.01). However, the relationship is even more significant if we take into account that the higher the number of tweets made for the hotel, the higher the number of neutral comments (Pearson correlation = 0.780, *p* ≤ 0.001) and negative comments (Pearson correlation = 0.688; *p* ≤ 0.001).

The coefficient of determination (R_2_) shows that 47% of the changes in the number of Tweets correspond to change in the number of negative tweets. The highest value of R_2_, 60.8%, is obtained by the number of neutral environmental tweets.

In addition, a descriptive statistical analysis was carried out using a difference of means test. It is quite frequent in market research to hypothesize whether the differences in behavior detected in two population subsamples give sufficient evidence for differences in the populations or if, on the contrary, they are only a product of sampling error. Before applying the mean difference test or Student’s *t* test, any statistically significant differences when comparing the averages of the two independent subsamples must be determined. 

The Levene Test must be performed to check whether the variances are different or not. A predictive model is said to present homoscedasticity when the variance of the error of the endogenous variable is maintained throughout the observations. Therefore, this condition is checked before carrying out the hypothesis test that leads to the *t* test. To do this, we divided the sample into two groups: 5-star hotels and 4.5-star hotels. As can be seen in Table 16, the difference in means was not significant, except in negative comments (*p* ≤ 0.05), where there are differences and where there are more negative comments in hotels with fewer stars.

## 6. Discussion

As the work of Honeycutt and Herring [50] indicates, the opinions and reviews of users can be analyzed from a consumer satisfaction perspective since concerns and opinions are identified regarding the purchase of a product, or use of a service [31,41]. Furthermore Agarwal et al. [35] indicates that an exploratory study can be carried out to link these concepts with any particular research theme [37]. In this way, this exploratory study links the main environment factors for the users who have visited hotels that appear in the Traveler’s Ranking 2017.

The work of Kwon [17] shows Sentiment Analysis as a research process that allows for correct classification of users’ opinions and reviews on social networks and 2.0 platforms. Social networks have given companies new ways to receive the impressions and expectations of their customers. The works of Rosa, Batista and Carvalho [42] and Halog [52] show the need to understand these new phenomena and above all to know how to identify problems and key factors. 

To do this in our exploratory study, key factors were identified that could help companies in the tourism sector to improve the quality of their services and offer better products [20] based on the improvement of environment factors identified in the investigation [7,13]. As stated, after conducting the Sentiment Analysis, a total of *n* = 14,459 tweets were identified for the sample of hotel users. These tweets were classified as negative, neutral and positive with an algorithm developed in Python and based on machine learning by MonkeyLearn API [7,26]. As we have already verified from the analysis of the research results by using a qualitative approach on the analyzed environment indicators, Twitter has been shown to be a valid platform for research on the environment.

This exploratory study shows that although some Twitter users make complaints or take advantage of offers and discounts, many users comment on the environmental factors which determined their expectations and experiences at hotels.

Therefore, this exploratory study demonstrates that it is possible to determine the relationships that hotel users in Spain have with the key environment factors that can be improved or attended to by hotels such as those related to activities carried out by the hotel and the impact of these on the environment, on air quality or on the pollution generated by these activities. This can be seen because the hotel users show interest in these factors in their tweets and so they consequently partially determine the satisfaction and experience that the visitors have when they stay at the hotels.

In addition, the quantitative analysis finds that a greater number of followers on Twitter does not mean that these followers are positive in their comments. In fact, the exploratory study shows that negative comments increase when hotels have more followers. This is due to the users who experience negative environmental factors usually follow the hotel’s Twitter profile to make complaints or indicate dissatisfaction with the services provided. Also, as a result of the quantitative analysis we saw that the number of followers on Twitter does not necessarily increase the number of positive comments of the hotel users in the Spanish hotel sector.

We also found that if the number of followers is greater, the neutral comments also increase which means that hotel users use Twitter as a source of information for activities, updates and monitoring of hotel communications, but not necessarily to express satisfaction with the hotel. Twitter has therefore consolidated itself as a social network that can be the object of exploratory study related to the environment. It can also help hotel managers to improve their services by identifying the environment factors which travelers have shown interest in during their stay.

## 7. Conclusions

Over the last few decades, the use of new technologies has led to changes in sectors related to environment, culture, sustainability and global warming [1,5]. The development of new technologies and especially social networks has allowed the tourism sector to incorporate important changes when assessing actions related to the environment and social responsibility policies [6,7]. For this investigation, the hotels which won the Traveler’s Choice Award, which is based on over 500 million travelers’ reviews on TripAdvisor, were used. These hotels have achieved ratings of between 4.5 and 5.0, with 5 being the highest valuation from people who used their services.

The findings of this exploratory study were analyzed to find the environment factors which could concern hotel users, with the negative, neutral and positive environment factors being classified as N_1_, N_2_ and N_3_. The affect that these environment factors have on the users who visit the hotels was also studied (N_4_) [7]. H1 is confirmed because after the textual analysis, it was seen that factors related to the environment influence the experience that users have in hotels, and these users refer to different factors based on their feelings with opinions and reviews of the hotels. H2 is accepted, as shown by the results of the exploratory study, because the N_1_, N_2_ and N_3_ structures have been developed and the main factors that affect hotel users have been structured and classified into three types of feelings; negative, neutral and positive. Likewise, H2 has been accepted since the results of the exploratory study show that there are connections between the reviews that users leave about their experiences in hotels on Twitter with the identification of factors related to the environment.

The contribution of this exploratory study is the identification of the key environmental concepts that can help the hotel industry to make improvements in the activities and actions related to the environment, significantly improving the perception that users have of topics related to ecology, traditional commerce, ecological products or air quality, among other factors. Hotels can use the results of this exploratory study to highlight the importance that the factors identified by users have for the hotel sector and act upon them. Therefore, the results of this exploratory study are relevant for hotel management because they can be used to add value to the strategies for improvement of tourist activities so that they respect the environment. Some examples such as hotel activities that could affect the garbage collection or atmospheric pollution in its surroundings. Also, improvements can be made in the hotel activities to support and respect local traditions, improve air quality, support traditional customs and ecology and respect and maintain activities that favor the natural environment of the geographical area in which the hotel is located.

This exploratory study shows positive and negative concerns about the quality of services when users travel and use the services and activities organized by the hotels. This shows that travelers really do care about the environment and the consequences of their trips. Spanish hotels can also use the results of this exploratory study to develop new strategies for corporate social responsibility and to correctly identify which environmental factors concern users so that hotel activities can be adapted to incorporate actions that support or disseminate local traditions and products, maintain the local environment and wildlife of rural areas, or respecting air quality in the areas where hotels have facilities and offer services. With the results of this exploratory study Spanish hotels can increase user satisfaction by taking into account the environmental factors that were identified in this study.

The limitations of this study are related to the number of subjects that make up the sample, the time frame in which the reviews were made and the qualitative analysis carried out to determine the topics of interest, as well as the Occurrence and Reliability percentages of the results.

Further studies could be made into the environment factors detected by hotel users and moderating variables such as age or sex, as well as the geographical areas in which the hotels are located. Also, how the users’ feelings about these factors influence their satisfaction with the hotel and its services, along with the real impact they have on the environment.

## Figures and Tables

**Table 1:** Tourism factors and their impact on the environment.

Factors	Environmental Impact
Accumulation of public	Stress for the environment and animals
Extreme sports	Disturb the fauna
Animal feeding	Changes in wildlife behavior
Diving and snorkeling	Damage to sea beds
Camping/Picnics	Erosion of the soil. Damage to vegetation. Noise. Rubbish
Hunting and fishing	Reduction of species
Off-road driving	Destruction of soil and vegetation
Noise emission	Disturb the animals
Climbing, hiking	Damage to vegetation
Cars	Running over animals, pollution, noise
“Souvenir” collection	Interruption of natural processes
Collecting wood	Deforestation, destruction of habitats
Throwing garbage	Deterioration of space and danger to local animal and human health
Discharge of waste not suitable for water	Water pollution, acidity
Construction of facilities	Loss and division of habitats
Construction of electricity pylons	Impact on birds in flight
Tourism infrastructure	Visual impact on fauna, vegetation and aquatic habitat

**Table 2:** International tourists depending on type of accommodation.

Type of Accommodation	Monthly Data	Yearly Variation	Accumulated Data	Accumulated Data
	Absolute Value		Absolute Value	Yearly Variation
TOTAL	3.979.713	13.3	75.563.198	10.3
Total paid accommodation (3)	2.841.464	15.1	59.419.138	11.7
—Hotel accommodation	2.299.988	11.2	47.726.623	11.2
—Rented housing	397.010	49.2	8.278.525	8.0
—Other paid accommodation	144.467	6.4	3.413.990	32.1
Unpaid accommodation	1.138.248	9.1	16.144.060	5.3
—Self-owned housing	320.539	42.5	5.039.040	15.7
—Relatives or friends house	704.622	0.3	9.550.942	9.4
—Other unpaid accommodation	113.087	−2.4	1.554.079	−31.1

**Table 3:** Arrival of international tourists according to access routes.

Access Routes	Monthly Data	Yearly Variation	Accumulated Data	Yearly Variation
	Absolute Value		Absolute Value	
TOTAL	3.979.713	13.3	75.563.198	10.3
Airport	3.197.756	16.6	60.582.406	11.7
Highway	696.586	0.1	13.038.391	4.4
Train	23.038	41.3	364.115	6.2
Puerto	62.332	8.8	1.578.287	9.6

**Table 4:** Characteristics of Sentiment Analysis in investigations.

Characteristics	References						
	Pak et al. [34]	Kuo et al. [9]	Honeycutts et al. [50]	Kouloumpis et al. [54]	Rodríguez-Herráez [41]	Boyd et al. [51]	This Research
Neuronal Connection	√	-	√	√	√	√	-
Textual analysis	-	√	√	-	-	√	-
Time	-	√	-	-	-	-	-
Hashtags, URLs or mentions	-	√	-	-	√	-	√
Topic	-	√	√	-	-	-	√
Classification of information	√	-	√	-	√	-	-

**Table 5:** Characteristics of textual analysis in investigations.

Characteristics	References							
	Kwon et al. [17]	Ramirez-Andreotta [18]	Rosa et al. [48]	Honeycutt et al. [50]	Boyd et al. [51]	Saito et al. [52]	Jiang et al. [53]	This Research
Classification into nodes	√	√	√	√	√	√	√	√
Categorization	√	√	-	-	-	√	√	√
Word Count	√	√	√	√	√	-	-	√
Key word	-	-	√	-	√	-	-	√

**Table 6:** Environmental Node classification with Nvivo software.

Factor Identification	Nodes
Negative	Node 1 (N_1_)
Neutral	Node 2 (N_2_)
Positive	Node 3 (N_3_)

**Table 7:** Environment data which was analyzed and average classification probability percentages for each Hotel.

Traveller’s Choice from TripAdvisor 2017	Tweets	Negative	Neutral	Positive	Average Probability
Hotel The Serras	159	1	90	68	0.639
Vincci Selección Aleysa Hotel Boutique & Spa	1665	81	734	850	0.610
Casa Camper Hotel Barcelona	52	1	39	12	0.555
Hotel Orfila	436	1	419	16	0.662
Hotel Abadia Retuerta Le Domaine	399	15	180	204	0.620
Gran Hotel Son Net	213	-	161	52	0.616
Hotel Maria Cristina	211	10	100	101	0.614
Only YOU Boutique Hotel Madrid	756	12	383	361	0.651
Alma Pamplona Muga de Beloso	40	1	16	23	0.674
Seaside Grand Hotel Residencia	128	58	938	726	0.624
Hotel Hacienda de Abajo	80	-	44	37	0.666
Hotel Olivia Balmés	35	-	28	8	0.609
Riviera Beachotel	369	9	225	136	0.682
Sant Francesc Hotel Singular	355	-	296	60	0.598
El Palace Hotel	373	19	173	182	0.600
Gran Hotel La Perla	1539	86	670	784	0.607
Barceló Emperatriz	1722	59	938	726	0.624
Hotel Astoria Playa Only Adults	93	1	62	30	0.596
Catalonia Square	2762	32	1859	935	0.652
H10 Cubik	1407	8	1214	185	0.711
Gold By Marina	720	19	398	304	0.622
Hotel Spa Relais	945	34	437	475	0.679
	*n* = 14,459				

**Table 8:** Results for N_1_ for environment factors identification.

N_1_	Count	Similar Factors	Weighted Percentage
Rubbish collection	16	rubbish, trash, waste	0.06
Atmospheric contamination	15	contamination, breathing, asthma	0.06
Pollution	14	dirty, dirt	0.06

**Table 9:** Results for N_2_ for environment factors identification.

Negative Tweets
1. Really disgusted by the unhygienic food that @barcelohoteles are serving at the #AllegroIsora really regret booking here for 8 days when staff won’t do anything about it.
2. @H10_Hotels we are at your resort in Punta Cana sick with food poisoning management does not care. Many guests are sick We will never return
3. @El_Felips paid for a upgraded room in Barcelo Lanzarote, so disappointed the room is tired, air con very poor, mould in bathroom and smelling very bad!! The installations and trips to locals places are also bad!

**Table 10:** Results for N_2_ for environment factors identification.

N_2_	Count	Similar Factors	Weighted Percentage
Local products	250	Streetmarket, markets, local produce	0.40
Handcraft	71	artisan, craftsmen, pottery	0.11
Traditional experience	67	shelters, seminars, monasteries	0.11
Looking after nature	57	rivers, villages, mountains, roads	0.09

**Table 11:** Neutral tweets examples.

Negative Tweets
1. RT @AnneSemonin We love to visit Hotel Sant Francesc for a spot of sunshine and a luxury Anne Semonin treatment.
2. RT @Stanatic_Ness: A beautiful street with so much of history and tradition #PalmaDeMallorca #Spain
3. Eating a local fish in #LaPalma @haciendadeabajo-medregal-tastes a bit like tuna, v tasty

**Table 12:** Results for N_3_ for environment factors identification.

N_3_	Count	Similar Factors	Weighted Percentage
Local Traditions	67	hostels, routes, local products, shows	0.29
Air Quality	32	clarity, pure, sight, breathing, asthma	0.12
Customs	44	dances, traditional production, pastry making, viticulture	0.21
Traditional Ecology	12	saltpans, orchards, eco-products	0.10
Nature	11	oasis, islands, mountain, rivers, trails	0.09

**Table 13:** Positive tweets examples.

Positive Tweets
1. #AQuintadaAuga is a #hotel surrounded by #nature in #SantiagodeCompostela. It is a mandatory stop
2. RT @AmyWorsley85: Always looking for a #sustainable place to stay, like the @Olivia_Balmes hotel in #Barcelona which was great!
3. @barcelohoteles @Bobadilla5GL Congrats! More hotels can be sustainable using local biomass for energy. Please visit us at @conectabioener!

**Table 14:** Results of N_4_ for environment factors identification.

Nodes	Environment Factors	Related Factors	Total Count
Positive Factors	Local Traditions	hostels, routes, local products, shows clarity, pure, sight, breathing, asthma, dances, traditional production, pastry making, viticulture, saltpans, orchards, eco-products, oasis, islands, mountain, rivers, trails	166
	Air Quality		
	Customs		
	Traditional Ecology		
	Nature		
Neutral Factors	Local products	street market, markets, local produce	445
	Handcraft	artisan, craftsmen, pottery	
	Traditional experience	shelters, seminars, monasteries	
	Looking after nature	rivers, villages, mountains, roads	
Negative Factors	Garbage collection	rubbish, trash, waste	45
	Atmospheric pollution	contamination, breathing, asthma	
	Pollution	dirty, dirt	

**Table 15:** Bivariate linear correlation results for environmental factors.

Correlation Results		Stars	Number of Environment Comments	Number of Environment Tweets	Number of Followers
Number of Followers	Pearson Correlation	−0.220	−0.183	0.588 **	1
	Sig. (bilateral)	0.326	0.415	0.006	
	N	22	22	20	22
Negative Tweets	Pearson Correlation	0.183	−0.325	0.648 **	0.371
	Sig. (bilateral)	0.414	0.140	0.002	0.089
	N	22	22	20	22
Neutral Tweets	Pearson Correlation	−0.152	−0.339	0.780 **	0.339
	Sig. (bilateral)	0.499	0.123	0.000	0.122
	N	22	22	20	22
Positive Tweets	Pearson Correlation	0.068	−0.303	0.688 **	0.381
	Sig. (bilateral)	0.764	0.171	0.001	0.081
	N	22	22	20	22

** Significance intensity.

**Table 16:** Differences of means test results.

Variable	F	Sig.	t	Difference of Means	Difference of Standard Error
Nº comments	1.363	0.257	−1.687	−510.933	302.795
Tweets	0.066	0.800	−0.414	−725.000	1750.570
Followers	2.943	0.102	−1.006	−16,259.150	16,157.287
Negative	7.454	0.013	0.833	9.683	11.620
Neutral	1.483	0.237	−0.689	−139.617	202.547
Positive	0.932	0.346	0.305	41.933	137.628

F: F Square; Sig.: Significance level; t: t-Student.

## Appendix A

**Table A1:** Winning traveler’s choice hotels from TripAdvisor sample in 2017.

Traveller’s Choice fromTripAdvisor 2017	Location	TripAdvisor Starts	Customer Reviews	Twitter Profile	Tweets	Followers
Hotel The Serras	Barcelona, Spain	5.0	843	@TheSerrasHotel	682	727
VincciSelecciónAleysa Hotel	Benalmádena, Spain	5.0	842	@Vincci_Hoteles	11.9 k	19.6 K
Casa Camper Hotel Barcelona	Barcelona, Spain	5.0	1924	@casacamper	1042	175
Hotel Orfila	Madrid, Spain	5.0	111	@HotelOrfila	726	356
Hotel AbadiaRetuerta Le Domaine	Sardón de Duero, Spain	5.0	449	@arledomaine	2642	1.365
Gran Hotel Son Net	Puigpunyent, Spain	4.5	598	@GranHotelSonNet	1260	3286
Hotel Maria Cristina	San Sebastián—Donostia, Spain	4.5	1794	@hotelmariacrist	1592	2218
Only YOU Boutique Hotel Madrid	Madrid, Spain	5.0	1975	@OnlyYOUHotels	4512	3877
Alma Pamplona Muga de Beloso	Pamplona, Spain	5.0	1187	@almahotels	68	86
La Bobadilla, a Royal Hideaway Hotel	Loja, Spain	5.0	774	@barcelohoteles	11.2 k	179 K
Seaside Grand Hotel Residencia	Maspalomas, Spain	5.0	653	@GrandHotelPunta	753	858
Hotel Hacienda de Abajo	Tazacorte, Spain	5.0	259	@HaciendadeAbajo	585	712
Hotel Olivia Balmés	Barcelona, Spain	4.5	2304	@Olivia_Balmes	548	7241
Riviera Beachotel	Benidorm, Spain	4.5	2314	@benidormhoteles	2912	1859
Sant Francesc Hotel Singular	Palma de Mallorca, Spain	4.5	476	@hotelstfrancesc	1490	1409
El Palace Hotel	Barcelona, Spain	4.5	1776	@ElPalaceHotel	2152	2512
Gran Hotel La Perla	Pamplona, Spain	5.0	336	@Ghotellaperla	6440	3020
Barceló Emperatriz	Madrid, Spain	4.5	568	@barcelohoteles	11.2 k	179 K
Hotel Astoria Playa OnlyAdults	Port d’Alcudia, Spain	4.5	2427	@astoriaplayasup	254	139
Catalonia Square	Barcelona, Spain	4.5	932	@CataloniaHotels	22.9 k	13.6 K
H10 Cubik	Barcelona, Spain	4.5	822	@h10hotels	10.9 k	18.4 K
Gold By Marina	Playa del Inglés, Spain	4.5	1596	@GoldByMarina	1.594	381
Hotel Spa Relais & ChateauxAuga	Santiago de Compostela, Spain	4.5	819	@aquintadaauga	4292	1996
Alma Barcelona	Barcelona, Spain	4.5	1876	@almahotels	68	86
Hotel Las Madrigueras Golf Resort and Spa	Playa de las Américas, Spain	5.0	305	-	-	-
